# Primary Malignant Melanoma of the Cervix: A Case Report and a Review of the Literature

**DOI:** 10.1155/2020/7206786

**Published:** 2020-09-03

**Authors:** Michail Diakosavvas, Zacharias N. Fasoulakis, Maria Kouroupi, Marianna Theodora, Lola Inagamova, Georgios Tsatsaris, Panagiotis Nikolaou, Konstantina Frangia-Tsivou, Alexandra Giatromanolaki, Emmanuel N. Kontomanolis

**Affiliations:** ^1^Department of Obstetrics and Gynecology, National and Kapodistrian University of Athens, Greece; ^2^Department of Pathology, Democritus University of Thrace, Greece; ^3^Department of Obstetrics and Gynecology, Democritus University of Thrace, Greece; ^4^HISTOBIO Diagnosis, Athens, Greece

## Abstract

**Background:**

Gynecologic melanomas are extremely rare malignancies, and primary malignant melanoma of the cervix (PMMC) is the rarest among them all, with less than 100 cases reported so far. Although some conditions have been correlated with the pathogenesis of this entity, no specific risk factor has been yet identified, with vaginal bleeding being the most common symptoms. The diagnosis is based on physical examination with speculum assessment and cytologic and histopathologic findings accompanied with immunohistochemical staining of lesion's biopsies. *Case Presentation*. We report a case of PMMC in a 34-year-old para-2 patient, among the youngest cases of PMMC reported, that presented to our clinic for routine examination. Gynecologic examination demonstrated a dark, heavily fully pigmented cervical growth completely covering the entire external cervical os. Biopsy obtained and showed malignant melanoma. She underwent radical hysterectomy with bilateral salpingo-oophorectomy and pelvic lymphadenectomy. The pathological diagnosis was FIGO stage IB1 PMMC. Despite 2 courses of anti-PD-1 antibody (Nivolumab) treatment, the patient passed away 13 months after diagnosis (12 months after surgery).

**Conclusions:**

Early diagnosis and subsequently early treatment are of high importance regarding patients' prognosis and survival. No standardized protocols or treatment guidelines specific for this rare cancer have been issued; thus, clinicians are called to assess each case individually. Current treatment options are based on surgical excision mostly with radical hysterectomy, but in advanced or recurrent state of the disease, other treatment modalities, such as chemotherapy, radiotherapy, and immunotherapy, can be employed. Prognosis for these patients is very poor, and survival rate remains extremely low, with the median OS reported being less than 2 years. Reporting and publishing of such cases are both of paramount importance for the better understanding of this uncommon cervical malignancy, and further biological and clinical investigations are required for more suitable and effective therapies to be determined. A new staging system, specific to PMMC, could be of great use for the better correlation of the disease's stage and prognosis of these patients.

## 1. Introduction

Cancer of the uterine cervix is one of the leading causes of cancer-related deaths in women worldwide [1]. Melanomas are malignancies that arise from melanocytes in the basal layer, most commonly of cutaneous origin [2]. Melanomas of the urogenital tract, which account for 3 to 7% of all mucosal melanomas, occur predominantly in the vulva (95%) and the vagina (3%) [3, 4]. Primary malignant melanoma (MM) of the cervix is one of the most rare malignancies among mucosal MM, with poor prognosis and with only a few cases reported in the worldwide literature [5]. The first well-documented case of primary malignant melanoma of the cervix (PMMC) was reported in 1944, by Taylor and Tuttle [6]. Since then, to this day, less than 100 cases of PMMC have been described [7, 8]. The diagnosis of PMMC can be proven very challenging due to the rarity of the disease and also due to the wide variety of cytomorphologic characteristics [9, 10].

In this study, we report a case of PMMC in a 36-year-old para-2 patient that presented to our clinic for routine examination and review the available literature regarding this entity, to identify the possible risk factors, the diagnostic approach, and the different treatment modalities that have been proposed.

## 2. Case Presentation

**Figure 1:**
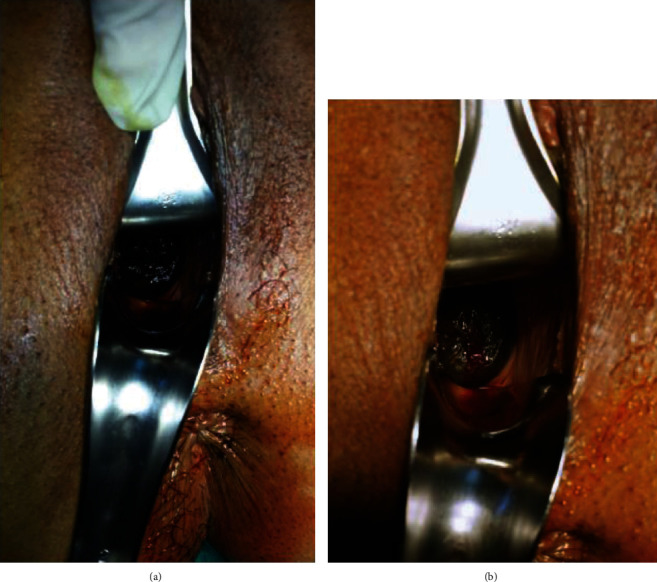
A fully pigmented growth was observable completely covering the entire external cervical os with smooth borders.

A 34-year-old female para-2 patient visited our clinic in 2012 for an annual examination. Her previous cervicovaginal Pap smear test was negative for intraepithelial lesions and neoplasia. The patient reported no previous medical history; she presented generally fit and well, only on medication for hyperthyroidism. Physical examination revealed a normal vulva, a vagina, and a dark, heavily fully pigmented cervical growth with smooth borders, completely covering the entire external cervical os (Figures 1(a) and 1(b)). The parametrium was not involved, and the growth was primarily restricted to the cervix. Incisional biopsy was performed, and the sample was sent for histopathological examination.

Macroscopically, there were three tissue fragments that range in pigmentation from dark to heavily melanized, and their diameter ranged from 0.4 cm to 1 cm. Multiple microscopic sections were characteristic of malignant melanoma. The tumor cells were epithelioid, with melanin in their cytoplasm (positive histochemical stain Masson-Fontana). Immunohistochemically, the neoplasm showed positivity for HMB-45, S-100, and Melan-A antigens, though it was negative for MNF116 antigen. Histopathology revealed a malignant neoplasm, which indicated the possibility of a primary cervical melanoma that was later confirmed.

Further clinical examination via MRI and PET/CT scan did not reveal any distant or lymph node metastases. A preoperative abdominal ultrasound examination was normal. A comprehensive assessment for melanotic lesions in the uveal tract (ophthalmoscopy), skin, and other mucosal sites was conducted, and the results were negative. The patient underwent radical hysterectomy with bilateral salpingo-oophorectomy and pelvic lymphadenectomy.

**Figure 2:**
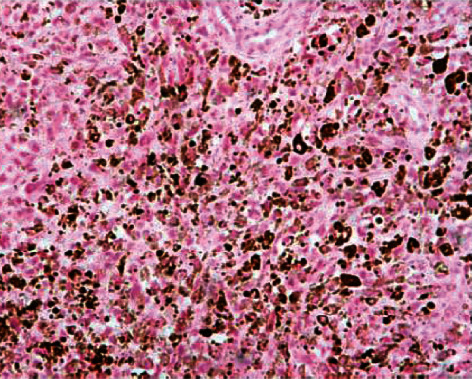
Tumor cells with characteristic melanin pigmentation. Haematoxylin-eosin, ×20 magnification.

**Figure 3:**
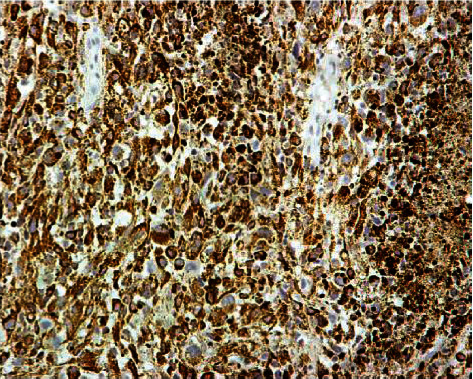
Immunochemical positivity for HMB-45, ×20 magnification.

The final diagnosis for the patient was stage IB1 melanoma (FIGO) of the cervix with no lymph node metastasis. The report also revealed a tumor mass consisting of medium to large sized neoplastic cells with markedly enlarged nuclei and discrete nucleoli with no clear cellular borders. Occasionally, giant cells could be seen (Figure 2). Immunohistochemistry reported diffuse positive reactions for HMB-45, S-100, and Melan-A. Melanoma tumor marker 5-SCD was 6.1 nmol/L (normal range 1.5-8.0) (Figure 3). Cytologic examination of the slides showed abundant large and round abnormal cells. They were either single isolated cells or gathered in aggregated groups. The solely cells had enlarged, hyperchromatic, and eccentrically placed nuclei.

A CT scan, 6 months later, was normal but, 9 months later, showed multiple hepatic and lung metastases, and she received 4 courses of anti-PD-1 antibody (Nivolumab) with the level of 5-SCD being elevated to 180 nmol/L. She passed away twelve [11] months after surgery.

## 3. Review of the Literature and Discussion

### 3.1. Incidence, Risk Factors, and Pathogenesis

Approximately 1% to 2% of all malignant melanomas (MMs) are mucosal, accounting for just 0.03% of all newly diagnosed cancers, with their most frequent site of origin being the head and neck (55%), anal and rectal regions (24%), and vulvovaginal region (18%) [2, 12]. A significantly small percentage (less than 2%) of primary mucosal melanomas of lower genital tract arise from uterine cervix, representing a very rare entity. Cervical MM's occurrence can be up to five times lower than primary MM of the vagina or vulva, and it is mainly diagnosed in postmenopausal women in the sixth or greater decade of their life with only 21.4% of the patients diagnosed being below 40 years old (median age 59.0 years) [7]. The patient treated in our institution was 34 years old at the time of diagnosis, and to the best of our knowledge, her age is among the youngest cases of PMMC ever reported and, possibly, even the youngest one.

Cutaneous MM's incidence has been increasing over the years which can be explained by the fact that they are associated with exposure to ultraviolent light. Conversely, mucosal MM's incidence remains stable, and since the anatomic location of such malignancies precludes ultraviolet exposure, no clear predisposing risk factors have been detected [11]. Nonetheless, irradiation should not be excluded as a possible risk factor, since Benson et al. reported in 2000 a case of radiation-induced cervical MM in a patient previously treated with radiotherapy for a squamous cell carcinoma of the cervix [13]. Moreover, the estrogen hormonal influence that can be anticipated after pregnancy and the HPV infection with subtype 16 that has been found in at least two cases of vulvar MM have been proposed as possible etiologic agents [9, 14, 15]. On the contrary, Nai et al. recently reported a case of a woman with PMMC postpartum who had used progestogen-based contraceptives after delivery, which was not related to the sex steroid hormone rise, since the tumor cells had been negative for estrogen and progesterone receptors. Instead, he suggested that the immunosuppression occurring normally in pregnancy may play a role as an oncogenic factor, and could be related to the proliferation of the tumor [16]. Furthermore, even though most cutaneous melanomas are closely related with NRAS and BRAF gene mutations, the same alterations are not equally detected in mucosal melanomas [16, 17]. Due to the rarity of the disease and the controversies reported, none of the risk factors, mentioned above, can be identified as specific; thus, safe conclusions cannot be reached.

For a long period of time, the existence of PMMC was questioned, based on the hypothesis that the cervix does not contain melanotic cells [18]. Their existence was not confirmed until 1959, when Cid discovered 3.5% melanocytes in the basal layer of cervical biopsies, proving that the development of MM in the uterine cervix was feasible [19]. These melanocytes could originate from Schwann cells, migrate from neural crest, or differentiate from the endocervical epithelium. [9] Their role in the cervical epithelium is not fully understood, but it is considered different from that of cutaneous melanomas [20]. Apart from PMMC, the melanocytes can give rise to other entities, such as cervical melanotic nevi and benign melanosis, a condition that has been proposed to be the origin of cervical melanomas [21, 22].

### 3.2. Diagnosis

#### 3.2.1. Clinical Manifestations

In the literature, there are a few asymptomatic cases that have been diagnosed on routine vaginal examination [7]. Likewise, the patient in our case reported no previous symptoms; thus, routine Pap smear examination was the reason for the initial cervical assessment and further diagnostic evaluation was ordered by the clinicians. However, most frequently, it presents as a symptomatic disease, since the cervix easily becomes ulcerated and infected [5]. The most common symptom is vaginal bleeding, the occurrence of which can reach up to 93% of cases [23]. Vaginal discharge, dyspareunia, abdominal pain, and postcoital bleeding have also been reported in some cases [9, 22, 24, 25]. Weight loss and hematuria, although less common, can also be present in MM of the lower genital tract [5, 7].

In the initial stage of the PMMC, the disease is contained to the cervical mucosa and, afterwards, spreads locally via direct dissemination to adjacent organs, such as the vaginal fornix, the uterosacral ligaments, the vulva, and the pelvic wall [26, 27]. Similarly, lymphatic metastasis, although rarely encountered at the time of diagnosis, follows the pattern of drainage of other cervical carcinomas [28]. Inguinal lymph nodes can also be involved in the case of vaginal wall participation [3]. Distant metastasis of PMMC is uncommon compared to cutaneous MM that have a high rate of metastatic spread. According to Pusceddu et al., only twelve cases of visceral metastasis have been described, with the most frequent sites being the liver peritoneum and bladder. Noguchi et al. described a case of PMMC which after the initial treatment progressed, and lung recurrence occurred [27]. Instead, cervical MMs seem to have a greater tendency towards regional spread [7].

#### 3.2.2. Diagnostic Approach and Pathology

The diagnosis of melanoma is based on physical history and clinical gynecologic evaluation with speculum assessment [23, 24]. It is usually determined by histopathologic examination of obtained biopsies with the use of colposcopy and by electron microscopy which is able to detect melanosomes and premelanosomes in the cells' cytoplasm and finally is confirmed by immunohistochemical staining mainly with S-100 and HMB-45 markers [18, 22, 27, 29].

#### 3.2.3. Cytology

In the past, the use of colposcopy and cytology has been employed for the early diagnosis of cervical melanoma [24]. Jin et al. successfully managed to put the diagnosis of PMMC in an asymptomatic patient with a history of supracervical hysterectomy through immunoreactivity to specific markers from a Papanicolaou smear test that showed high-grade dysplasia (CIN3) [30]. Üzüm et al. were the first to report a diagnosis of cervical melanoma using liquid-based cytology after colposcopy, despite the fact it was concerning a metastatic lesion of cervical MM, without any gross cervical pathology [31]. Fine needle aspiration cytology has also been used as a tool in the diagnostic process of PMMC [32].

#### 3.2.4. Histology

*(1) Macroscopic Examination*. Cervical MM is presented with diverse morphology and a wide spectrum of macroscopic features which range from exophytic, fungating, or polypoid cauliflower-shaped mass with visible pigmentation and clear signs of atypia to single or multiple well-defined plane or ulcerated nodules [5, 22, 23, 33–35]. The size of the tumor may be 0.3 to 9 cm (mean diameter 3 cm), as was in our case [7]. The color of the lesions varies from dark black-brown-blue, due to melanin accumulation, to reddish and in some cases pinkish [7, 34, 36, 37]. It should be mentioned that melanin is not always detected in cervical MM cells and approximately 45% of these tumors are amelanotic, compared to 10% of cutaneous amelanotic MMs [26]. The absence of melanin is a characteristic feature of mucosal melanoma, which can make the distinction between MM and lymphoma or angiosarcoma difficult [38]. Benign melanosis of the cervix and cervical endometriotic nodules could also be encountered and could be misdiagnosed as MM since they may also appear dark brown [35].

*(2) Microscopic Examination*. The spectrum of cellular features is broad; hence, lesions may involve spindle cells with intracytoplasmic coarse melanin particles, groups of large cells with hyperchromatic nuclei, and prominent nucleoli inside the eosinophilic cytoplasm or large pleomorphic cells, consisting of great cytoplasmatic area, large vesicular nuclei, and notable abnormal mitoses [7, 23]. High mitotic index and cell pleomorphism are commonly encountered [7]. Findings similar to the ones observed in cytologic samples are the intranuclear cytoplasmic inclusions, hyperchromatic nucleoli, and multinucleation of giant cells [7, 39, 40]. The tumor, which consisted of a large vasculature network, usually infiltrates the cervical mucosa and exhibits signs of bleeding, necrosis, and inflammatory response. The stroma in many cases is presented desmoplastic, while junctional changes as well as a pagetoid pattern of intraepithelial spread of tumor cells may be identified in the adjacent epithelium [7, 35].

Based on a study regarding melanomas of the vaginal walls, Norris and Taylor have published criteria that have been established and adopted by the majority of the clinicians and pathologists for the diagnosis of primary MM of the cervix. Nonetheless, in many cases of PMMC, these criteria are not completely fulfilled, as mentioned by the same authors [7–9, 33]. Recently, in a study by Yin et al., the following criteria were suggested specifically for the diagnosis of PMMC: (i) pigmentation in the normal cervical epithelium, (ii) absence of melanoma anywhere else in the body, (iii) a clear junctional change in the cervix, (iv) a pagetoid intraepithelial component, (v) complete absence of CK/epithelial immunohistochemical findings, and (vi) metastases that follow the pattern of cervical carcinoma [23].

*(3) Immunohistochemical Examination*. Markers that are commonly positively expressed are S-100, HMB-45, Melan-A(MART1), Vimentin, and SOX10 [5, 7, 23, 35, 37]. Negative expression of Cytokeratin is being observed, similarly to cutaneous MMs [33, 36]. S-100 is considered the standard marker, with high sensitivity for primary and metastatic MM, that reaches up to 94% [23, 27]. HMB-45 is also reliable due to its high specificity for MM, despite its sensitivity ranging between 60 and 80% [23]. The combination of both appears to offer the best results, since it can discriminate amelanotic melanoma from other tumors [7, 37]. Melan-A also demonstrates high sensitivity and specificity [23]. Similarly, as previously mentioned, in our case, the tumor revealed positive expression of markers S-100, HMB-45, and Melan-A. Nonetheless, frequently, HMB-45, and Melan-A may be expressed weakly or negatively in desmoplastic variants of melanomas or spindle cells [35]. Ki-67 is another marker that is employed in order to distinguish a benign melanoma mole (<5%) and MM (>25%) [23]. In the literature, the use of several other markers of the melanocyte line has been reported, including NK1/C-3 and NK1 BETEB [20]. Electron microscopy can be employed since it is able to detect melanosomes and premelanosomes in the cells' cytoplasm [29].

#### 3.2.5. Differential Diagnosis

The establishment of PMMC diagnosis requires the exclusion of metastatic MM, from another primary neoplastic site, which is mainly of cutaneous, but may also be of mucosal origin [7, 41]. Thus, the skin, the uveal tract, the esophagus, and the anorectal region should be promptly investigated for a primary lesion [33, 36]. The absence of junctional activity on histology could be an indicator of a possible metastatic rather than primary MM of the cervix [33]. However, it should be mentioned that differential diagnosis of primary vs. metastatic lesion of the cervix may not be feasible, especially in cases of advanced neoplastic processes and ulceration, which may cause junctional changes [7]. When the cervix is secondarily affected, the neoplastic cells tend to be localized below the basal membrane which renders the detection of the junctional activities impossible [7]. Conversely, the presence of the malignancy in the cervical mucocutaneous transition area between squamous and columnar epithelia could be a sign of PMMC, since this is where primary MMs are most commonly observed [38]. Metastasis to the uterine cervix is considered extremely rare, due to the limited blood supply and the cervical fibrous stroma, which is an unfavorable environment for metastatic lesions to grow [18]. It has been reported that although the cervix is usually the origin of MM, almost 20% of all cervical MM are metastatic [22]. Secondary involvement of the cervix from a MM occurs either with locoregional spread of vaginal/vulvar carcinomas or with hematogenous metastasis of a distant primary MM [8, 42]. Multifocal presence of melanoma foci in multiple sites can prove the diagnosis very challenging. Although cutaneous MM can be multifocal in 5% of the cases, mucinous MMs can present in this state with an incidence of 20% [10]. Akoz et al. presented a case of synchronous cervical and vulvar MMs, and when confronted with the dilemma of origin of the malignancy, they concluded that since vulvar MM was detected on vulvar biopsy, and the cervical MM was only found during preoperative examination, the local recurrence of a vulvar metastasis was the most probable scenario [42]. On the other hand, in another case report from Pang et al., where the same synchronous MMs were being met, the exact primary site of origin—cervix or vulva—could not be determined. Hence, this is considered the first case ever being reported, concerning such a rare synchronous involvement of the cervix and vulva from a MM [8].

Moreover, PMMC should be differentially diagnosed from other entities of which it can be easily confused [10]. In the past, PMMC, mainly amelanotic, has been misdiagnosed as poorly differentiated squamous cell carcinoma or malignant peripheral nerve sheath tumor [43, 44]. Therefore, it is of high importance for other cervical malignancies to be considered in the differential diagnosis of PMMC, especially in the absence of pigment; and these are adenocarcinomas, rhabdomyosarcomas, leiomyosarcomas, mixed Mullerian tumors, large cell neuroendocrine carcinomas, anaplastic lymphomas, stromal sarcomas, undifferentiated carcinomas, clear cell carcinomas, and malignant peripheral schwannomas [9, 23, 27, 31, 33, 45]. It must be noted that the rest of the spectrum of melanocytic lesions, including blue nevi, which present flat or slightly raised, blue to black, and with unclear boundaries, and benign lentigines should also be included in the differential diagnosis of PMMC [18, 23]. Immunostaining can be proven extremely useful for the diagnosis of MM for most of these cases [5].

#### 3.2.6. Staging

Once the diagnosis of PMMC has been confirmed, further evaluation is required for the detection of possible metastatic sites and the complete staging of the disease. This includes a brain-chest-abdomen-pelvis computed tomography scan (whole-body CT) or a positron emission tomography (PET/CT) and serum lactate dehydrogenase (LDH) analysis [2, 7, 25, 46]. Magnetic resonance image scan (MRI) may also be employed, since it can distinguish melanoma from other tumors due to a distinct signal pattern for melanin (high signal intensity on T1-weighted image and low signal intensity on T2-weighted images) [4, 34].

The depth of the lesion's invasion (Breslow system) is an important prognostic factor in primary cutaneous melanomas [24]. Because the majority of cases with mucosal melanoma frequently present as large polypoid masses, this system is of little use in disease staging [2]. As a result, the International Federation of Gynecology and Obstetrics (FIGO) staging system has prevailed among authors and has been widely applied, since it correlates better with prognosis and survival and given the similar clinical presentation and pattern of spread of PMMC and cervical adenocarcinoma [5, 8, 24, 28, 34, 37, 47].

Pusceddu et al., in the largest analysis known to this day, which included 78 cases of published cases of PMMC, found that according to FIGO, stage I accounted for 41% of the cases, stage II for 34.4%, and stage II and IV for 18.0% and 6.5%, respectively [7]. Yin et al. also investigated the correlation between percentage and FIGO stage of PMMC in 44 published cases, including their cases reported, reached to comparable results, with 50% of the cases being classified as FIGO stage I and 32% as stage II [23]. However, the prognosis and survival rate of the patients in the analyses mentioned above were extremely low, despite the recognition of the disease at an early stage in about 80% of the cases.

Considering the increasing amount of reported data, nowadays, a new staging system specific to PMMC could be of great use and should possibly be created, in order to be more useful and suitable, regarding the correlation between patients' stage and prognosis.

### 3.3. Treatment and Prognosis

#### 3.3.1. Therapeutic Approach

Most therapeutic schemes that have been used over the years, in different case reports, are unpublished, and the treatment applied was based on that of cutaneous MM [3].

Therapeutic approach is not standardized but, instead, is individualized based on the characteristics of the disease and the patient [38]. The most commonly recommended therapy for PMMC is surgical excision followed by focal external beam or intracavitary radiotherapy (RT). Adjuvant treatment measures such as chemotherapy (CHT), RT, biotherapy, and immunotherapy are usually applied in selected cases of metastatic disease and locoregional recurrences targeting to palliate the symptoms and systematically control the disease [4, 48]. Different treatment schemes have been suggested, which consisted of neoadjuvant CHT and radical surgery followed by chemoradiation, but they were not widely applied, due to lack of prospective, randomized, clinical trials [49]. The absence of such studies assessing the effectiveness of different therapeutic options that have been applied in patients with gynecologic melanoma may be attributed to the rarity of PMMC cases [37].

#### 3.3.2. Surgical Treatment

Due to the lack of recommendations and specific guidelines, most patients with PMMC are usually treated according to the treatment protocols established for cervical squamous cell carcinoma [47]. Surgical treatment usually consists of total or radical hysterectomy with or without bilateral salpingo-oophorectomy (RAH/TAH+BSO), usually combined with or without pelvic lymphadenectomy (LND) and/or partial-superior vaginectomy (PV). In some cases, only local excision has been used [7]. However, it is argued that RAH results in better outcome regarding overall survival (OS) than TAH, based on a report of 14 cases of PMMC (66.8 months VS 19.5 months, *P* = 0.016) [28]. The main concern, as noted, should be the negative margins postoperatively; nonetheless, some authors recommend clean surgical margins of at least 2 cm, due to the aggressiveness of the disease [38, 47, 50]. Pelvic lymphadenectomy (PL) remains a subject of controversy. Some authors and clinicians advocate its use in low-risk surgical patients or in cases with large growth of the tumor with the presence of pigmented lymph nodes since it offers a potential benefit in the survival and allows for a more accurate staging of the disease in order to plan for any adjuvant therapy [18, 28, 36]. It has also been reported that in cases of bulky unresectable disease, a palliative and more conservative surgical option is suggested, with local and limited excision of lesions identified [7, 28].

#### 3.3.3. Radiotherapy

Results in patients treated with RT have demonstrated a poor prognosis regarding the survival [7, 51]. Melanoma is considered a radio-resistant malignancy; thus, the role of RT in the treatment of gynecological MM is limited and is mostly considered an adjuvant, preoperative, or palliative treatment [23]. The cases where it is usually employed are the recurrences, advanced disease, unsatisfactory surgical margins after initial operative approach, residual tumors, and pelvic lymph node involvement [24, 37]. The combined use of external RT and brachytherapy has not been well investigated [7]. Conversely, Shrivastava et al. reported that RT may reduce local recurrence in approximately 25% of the patients and is considered the main modality of treatment [47].

Recently, carbon-ion radiotherapy (C-ion RT) has been investigated as an advanced modality for the treatment of gynecologic MM, unfortunately not specific to PMMC. The results for 3-year local control and OS rates were 49.9% and 53%, respectively, and the 5-year estimated OS was 28%, while none of the patients developed late grade 3 or worse adverse effects and toxicities. Given that a therapeutic effectiveness similar to that of surgery was achieved, the authors suggest that C-ion RT could be an alternative choice to radical treatment for gynecologic MM, although more research is needed to validate the beneficial effect of this therapeutic agent [52, 53].

#### 3.3.4. Chemotherapy

In cutaneous MM, chemotherapeutic agents such as dacarbazine have been shown to reduce the size of the tumor with a response rate of about 15-20% when they are applied in cases of advanced disease of gynecological MM [27]. With this rationale, the same protocols have also been administered to patients with PMMC, in combination with cisplatin, vincristine, vinblastine, and bleomycin [8, 47]. Liu et al. reported a case of PMMC FIGO stage IB1 treated with this combination in conjunction to surgery, which remained disease-free for a minimum of 29 months, posttreatment [18]. Interferon A has also been used as an adjuvant CHT agent with promising results, given that the patient it was applied to achieved disease-free interval of 50 months after the surgery [8]. Nevertheless, the usefulness of CHT approach remains controversial, since no specific agent has proven efficient enough in reducing the rate of recurrence and since most data are derived from anecdotal experience [7].

#### 3.3.5. Immunotherapy and Biotherapy

The role of immunotherapy and biologic-response modifiers in cases of PMMC is still controversial. Ipilimumab, a monoclonal antibody targeting cytotoxic T lymphocyte antigen-4 (CTLA-4), has been reported to positively affect OS rate with researchers suggesting its use in conjunction to other CMT agents including antibodies targeting programmed death-ligand 1 (PD-1), BRAF, KIT, VEGF, and MEK1/MEK2 mutations such as Nivolumab, Vemurafenib, Dabrafenib, Imatinib, Bevacizumab, and Trametinib, respectively. Moreover, trials studying the use of immunotherapy, using local bacille-calmette-guerin (BCG) or activated lymphocyte transfusions, alone or combined with radiation, have supported the positive effect of immunotherapeutic schemes in PMMC [28, 54]. Hodi et al. compared treatment with Ipilimumab to a glycoprotein peptide vaccine in a phase 3 study of metastatic melanoma reporting an improvement in median OS of 10 months, as compared to 6.4 months in patients receiving the vaccine alone [55]. In addition, Schiavone et al. reported a study where a patient was treated with immunotherapeutic agents, followed by external beam radian therapy (EBRT) to 3000 cGy in 5 fractions, and underwent hysterectomy with BSO and upper vaginectomy 97 days after completing treatment, with promising results, receiving Pembrolizumab for recurrent disease after 19 months of initial diagnosis [56]. However, even though such studies have reported promising results considering OS, supporting its possible use as adjuvant therapy preoperatively or intermittently in prolonged therapies, many studies question its actual benefits. Pinedo et al. reported the use of BCG, interferon (IFN), and interleukin 2 (IL-2) in two patients with PMMC, with poor results [57]. In addition, Kim et al. treated a patient with PMMC with radical hysterectomy, upper vaginectomy, bilateral salpingo-oophorectomy, and pelvic lymphadenectomy that underwent 2 cycles of adjuvant immunotherapy with Pembrolizumab postsurgery but died within 8 months of the initial diagnosis [58].

#### 3.3.6. Prognosis and Survival

Cervical malignant melanoma is a particularly aggressive type of cancer with a very poor long-term prognosis, as shown in a clinicopathologic and molecular analysis of nonvulvar melanomas of the female genital tract, since both local recurrence and wide spread metastases usually occur within a short period from initial diagnosis (approximately between 2 months to two years) [17]. As a matter of fact, the patient in our case, despite the early stage of the disease at the time of diagnosis (FIGO IB1) and the surgical treatment with RAH+BSO+LND, developed multiple distant metastasis in lungs and liver, just 9 months after the initial diagnosis, and unfortunately passed away twelve months postoperatively.

Based on an analysis of 78 patients, concerning different therapies, the authors recommended surgery alone for stage I cancer and multimodal treatment with surgery and chemotherapy and/or radiotherapy for advanced stages [7]. Regardless of stage and treatment and despite the fact that almost half of the cases presented in the literature were diagnosed at FIGO stage I disease, the average survival reported (87.5% of patients presented) is less than 36 months of diagnosis (22.9 months overall mean survival, 12 months median OS, range 0.1-168 months) [59].

In a review of thirteen patients with PMMC, Lee et al. reported a 25% 5-year survival rate for stage IA, 14% for stage II, and 0% for stages III and IV [34]. Yuan et al. described a median OS of 33 months for stage I, 24 months for stage II, and 10 months for stage III among 14 patients with PMMC [28]. In another study, 5-year survival of patients with cervical melanoma was reported to be approximately 10%, and prognosis was mainly correlated with tumor stage at the time of diagnosis, even though most patients are, as mentioned, diagnosed at an early stage [60]. Thus, considering the prognosis of PMMC, due to the rarity and the diversity on the stage of the different cases reported worldwide, five-year OS ranges from 18.8 to 25% for stage I, 11.1 to 14% for stage II, and 0% for stages III–IV [7].

## 4. Conclusions

Early diagnosis and subsequently early treatment are of great importance regarding patients' prognosis and survival. No standardized protocols or treatment guidelines specific for this rare cancer have been issued; thus, clinicians are called to assess each case individually. A multidisciplinary team consisting of gynecologist-oncologists, medical oncologists, histopathologists, and researchers should collaborate, when this cancer is met, and each case should be discussed thoroughly for the patient's best possible therapeutic approach. Current treatment options are based on surgical excision mostly with radical hysterectomy, but in advanced or recurrent state of the disease, other treatment modalities, such as chemotherapy, radiotherapy and immunotherapy, can be employed, although some authors suggest their use, even in earlier stages. The prognosis of these patients is very poor, and survival rate remains extremely low with the median OS being reported less than 2 years, despite the fact that the disease is diagnosed mostly at an early stage. Therefore, we suggest that a new staging system, specific to PMMC, should possibly be created by the relevant scientific societies for the better correlation of the disease's stage and prognosis of these patients.

Most published studies consist of case reports or small case series, and only a few authors have tried composing an analysis, due to the disease's rarity (less than 100 reported cases, including ours). Nonetheless, because of this low incidence, research and literature are scarce and insufficient; hence, this leads to controversies and possibly inaccuracies in practices applied by clinicians across the globe. Reporting and publishing of such cases are of paramount importance for the better understanding of this uncommon cervical carcinoma, while further biological and clinical investigations are required for more suitable and effective therapies to be determined.
